# Studying the Adsorptive Behavior of Poly(Acrylonitrile-*co*-Styrene) and Carbon Nanotubes (Nanocomposites) Impregnated with Adsorbent Materials towards Methyl Orange Dye

**DOI:** 10.3390/nano11051144

**Published:** 2021-04-28

**Authors:** Khamael M. Abualnaja, Ahmed E. Alprol, M. A. Abu-Saied, Abdallah Tageldein Mansour, Mohamed Ashour

**Affiliations:** 1 Department of Chemistry, College of Science, Taif University, P.O. Box 11099, Taif 21944, Saudi Arabia; k.ala@tu.edu.sa; 2 National Institute of Oceanography and Fisheries, NIOF, Cairo 11516, Egypt; microalgae_egypt@yahoo.com; 3 Polymeric Materials Research Department, Advanced Technology and New Materials Research Institute, City of Scientific Research and Technological Applications (SRTA-CITY), New Borg El-Arab City 21934, Egypt; mouhamedabdelrehem@yahoo.com; 4 Animal and Fish Production Department, College of Agricultural and Food Sciences, King Faisal University, P.O. Box 420, Al-Ahsa 31982, Saudi Arabia; 5 Fish and Animal Production Department, Faculty of Agriculture (Saba Basha), Alexandria University, Alexandria 21531, Egypt

**Keywords:** methyl orange removal, water treatment, polymeric (acrylonitrile-*co*-styrene), multiwall carbon nanotubes, isotherm

## Abstract

In this study, a polymeric (acrylonitrile-*co*-styrene) P(AN-*co*-St) composite was impregnated with adsorbents, such as sulfonated and multiwall carbon nanotubes (MWCNTs), to increase the adsorptive characteristics of the nanocomposite upon the removal of methyl orange (MO) dye under different conditions. A novel nanocomposite copolymer mixture of P(AN-*co*-St) and SP(AN-*co*-St) was used. MWCNTs were prepared by a low-cost chemical vapor deposition (CVD) process. Variation in MO adsorption onto the three nanocomposites was examined in an aqueous solution via the batch technique with respect to contact time, initial MO concentration, adsorbent dosage, pH, and temperature. The surface of the nanocomposites was characterized by a scanning electron microscope (SEM), particle size distribution (PSD), Fourier transform infrared (FTIR), and Raman analysis. The experimental data showed that the efficiency of P(AN-*co*-St)/ MWCNT removal increased under the conditions of an acidic pH (3 and 5) with an agitation speed of 140 rpm, a sorbent weight of 0.01 g, and 20 mg of initial dye. The maximum sorption capacities were 121.95, 48.78, and 47.84 mg g^−1^ for the P(AN-*co*-St)/ MWCNTs, SP(AN-*co*-St), and P(AN-*co*-St) composites, respectively, as assessed by the Langmuir model. Additional isotherm models, such as the Freundlich, Temkin, and Halsey models, were used to examine the experimental data. A pseudo-second-order model was found to be more fitting for describing the sorption.

## 1. Introduction

Wastewater effluents comprise a variety of toxic and organic materials that are unsafe for fish and aquatic organisms [1,2]. Wastewater from dyeing is released into the environment and is a consequence of rapid industrialization. This wastewater originates from cosmetics, printing dye activities, leather paper, plastics, rubber, food processing, tanning, and textiles [3] and represents a major global concern. Most dye compounds are complex organic particles that are resilient against numerous factors, such as the action of cleaners and detergents [4]. Therefore, it is desirable to eliminate dyes from industrial effluents [5]. Techniques used in the removal and purification of dye-bearing wastewater effluents include electrocoagulation, chemical precipitation, photocatalytic degradation, oxidation, adsorption, and ozonation [6]. However, adsorption is considered to be superior to other methods. This is due to its inflexibility to a wide range of dyes, low cost, design simplicity, being biodegradable, ease of operation, and readily obtained adsorbents [7,8]. Nevertheless, its effectiveness is mostly based on the affinity of the adsorbate to adsorbent materials [9]. Solid–liquid adsorption techniques represent the most economical process for the removal of dyes from industrial effluents as other techniques are not efficient for whole decolorization, consume high energy, and require expensive equipment [10,11]. There are numerous polymeric compounds with functionality appropriate for application in the process of dye removal [12]. Poly (acrylonitrile-*co*-styrene) (P(AN-*co*-St)) is an excellent copolymeric adsorbent characterized by thermal and chemical stability, high compatibility with polar substances, and the simplicity of obtaining stabilized products due to the ladder structure created by nitrile polymerization [13]. 

In addition, molecular chains of P(AN-*co*-St) hold a cyano group, which may be improved. It can be hydrolysable and adjusted to realize functionality in a number of applications. It has been used for the treatment of heavy metal and dye wastewaters, as reported by [1,3,10,14,15]. Poly acrylonitrile was used by Elkady et al. [16] for removal of basic violet dye from aqueous solution, which revealed a good performance in the treatment of dyes, with a maximum adsorption capacity recorded at the level of 67.11 mg g^−^^1^.

Furthermore, carbon nanocompounds have attracted wide interest in the adsorption of pollutants [12]. Specifically, multiwall carbon nanotubes offer significant improvements in the sorption of dyes due to the high surface area of their caved nanocompounds, strong mechanical properties, and their excellent chemical stability, which make them necessary for dye adsorption [17]. The adsorption mechanisms of organic materials on carbon nanocompounds can function through the interplay among various intermolecular forces such as hydrogen bonding, π–π bonding, van der Waals forces, electrostatic interactions, and hydrophobic interactions, whose influences are based on the nature of the adsorbate and the surface chemistry of the adsorbent [5,18]. 

MWCNTs have a great potential for the elimination of dye pollution, as reported by Mohammed et al. [19], Machado et al. [20], Yao et al. [21]. In addition, Zare et al. [22] studied the elimination of noxious Congo dye by MWCNTs from aquatic solutions and concluded that it has an excellent potential of 92%, with a q_max_ of 352.10 mg g^−1^ of dye. Recently, Yu et al. [23] used carbon nanocompounds for the removal of methylene blue and congo red dyes from aqueous solution. The maximum capacity of the adsorption process was found to be methylene blue and congo red at the levels of 1387.2 and 351.7 mg g^−1^.

In current study, novel sulfonated poly (acrylonitrile-*co*-styrene) and poly (acrylonitrile-*co*-styrene) nanocomposites were prepared via a simple precipitation polymerization process, and multiwall carbon nanotubes were produced by the CVD process. The impregnation effect was demonstrated for sulfonated and multiwall carbon nanotubes into the P(AN-*co*-St) nanocomposite at a 1:1 ratio for the removal of methyl orange dye from the aqueous solution. The characterizations of the three copolymers were confirmed through scanning electron microscopy (SEM), Fourier transform infrared (FTIR) analysis, particle size distribution (PSD), and Raman spectroscopy. The suggested mechanism for the adsorption process was examined via kinetic and isothermal models. 

## 2. Materials and Methods

### 2.1. Materials

Methyl orange (2-(N,N-dimethyl-4-aminophenyl)azobenzenecarboxylic acid), used as a commercial dye, was obtained from the Central Drug House (P), Ltd. (Delhi, India), and this dye was used without additional purification. This methyl orange had been prepared through the diazotization of anthranilic acid followed by a reaction with dimethyl aniline. The chemical formula, molar mass, types of dyes, density, melting point, molecular structure, and extreme adsorption wavelengths are shown in Table 1. The prepared stock mixture was stored in the dark to avoid exposure to direct light. P(AN-*co*-St) was prepared in a 50:50 (*v/v*) solution of various monomer molar ratios of acrylonitrile. MWCNTs were produced by a (CVD) process using tube furnace through a 60 cm length quartz tube with a 45 mm diameter. Ethanol, sulfuric acid and nitric acid were acquired from International Co. for Supp. & Med. Industries (Cairo, Egypt). Acrylonitrile (AN) and Styrene (St) were obtained from Sigma Aldrich (Steinheim, Germany). NaOH and HCl were purchased from Nasr Pharmacetic Chemicals (Cairo, Egypt).

### 2.2. Chemical Reagents

A stock solution of methyl orange was prepared by dissolving 1 g of methyl orange powder in 1 L of distilled water. All the reagents and chemicals used in this work were of analytical grade, and each solution used in the adsorption experiments was prepared by diluting the stock mixture to the necessary concentration. The solution pH was adjusted using 1 M HCl or 1 M NaOH. 

### 2.3. Preparation of Adsorbents

#### 2.3.1. Preparation of P(acrylonitrile-*co*-styrene) Nanocomposites

The simple precipitation polymerization technique was used to prepare P(AN-*co*-St) with a 1:1 acrylonitrile (AN) and styrene (ST) solvent. The copolymerization method used distilled water as a cosolvent and ethanol as a solvent, followed by the stepwise injection of the initiator 0.01 M potassium persulfate (K_2_S_2_O_8_) at 55 °C for 4 h [1]. After completing this step, the polymer was separated by centrifugation at high speed (14,000 rpm) and then washed numerous times with an ethanol–distilled water mixture to remove any unreacted monomers or excess initiators. The polymer was isolated via centrifugation at a high speed (14,000 rpm) and successively washed with an ethanol–distilled water mixture at 55 °C. Then, the collected polymer was dried at 70 °C in an oven overnight. Characterization of the obtained white powder compound was used to confirm the identity of the product [7].

#### 2.3.2. Preparation of Sulfonated P(AN-*co*-St) Nanocomposites

**Figure 1:**
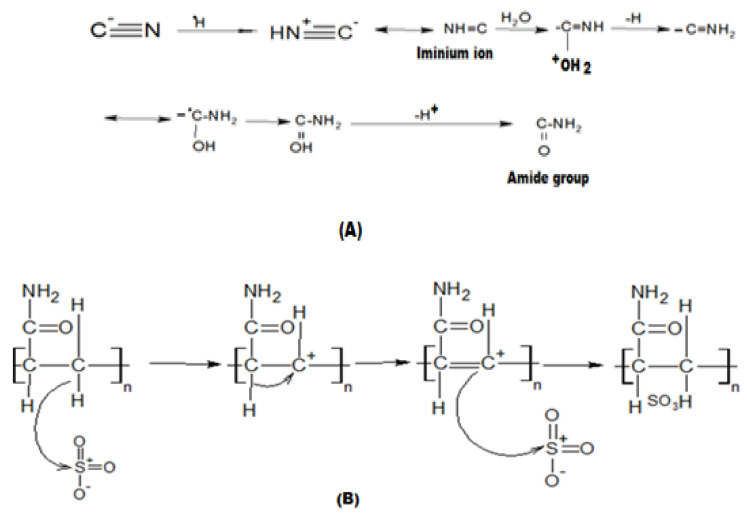
Mechanism of reaction for (**A**) hydrolysis of the CN group and (**B**) SP(AN-*co*-St).

P(AN-*co*-St) was dried at 100 °C in vacuum for 24 h, and 3 g of P(AN-*co*-St) was then melted in 300 g of conc. H_2_SO_4_ (98%) and agitated at 100 °C for 72 h using silicon oil (as a bath). The mixture was then cooled to room temperature and precipitated in ice water under a constant agitation rate. Over a few hours, the reddish suspension was allowed to settle. Next, the filtrate and the polymer were washed with distilled water until the pH value of the washed water was neutral. The precipitated polymer was then dried in a vacuum oven at 100 °C for 24 h with AN with a 12% degree of sulfonation (DS) and the requisite 72 h reaction time at 100 °C under mechanical agitation [21] (Figure 1B). 

#### 2.3.3. Preparation of MWCNTs

MWCNT synthesis was carried out using the chemical vapor deposition technique (CVD). This method can be scaled up to prepare amounts of MWCNTs suitable for manufacturing according to Bahgat et al. [24] and Eldeeb et al. [25].

### 2.4. Purification and Functionalization of MWCNTs

Surface functionalization helps to steady the dispersion as this technique can inhibit the reaggregation of nanotubes and be used for coupling of the polymeric matrix via MWCNTs. The functionalization of MWCNTs could be realized by determining various functional groups on the surface sites of the MWCNTs via oxidizing agents, such as acids that are produced during the creation of hydroxyl groups or carboxylic (–COOH, –OH) groups on the surfaces of MWCNTs, which is known as the defect group functionalization type. The functionalization method was achieved as reported by Eldeeb et al. [25], where 10 mL of concentrated sulfuric acid and 30 mL of nitric acid were placed in a flask loaded with 10 g of the prepared MWCNTs and 5 g phosphorous pentoxide. The mixture solution was refluxed for 120 min at 350 °C to obtain the MWCNT suspension mixture [24]. The mixture solution was then washed using deionized water followed by drying at 50 °C overnight to obtain carboxylate MWCNTs.

### 2.5. Batch Adsorption Experimental Run

The adsorption trials were investigated in batch equilibrium mode using an aqueous methyl orange solution. The effect of varying adsorbent dose (0.001–0.1 g), initial MO concentration (20–100 mg L^−1^), pH (3–11), adsorption temperature (30–60 °C), and contact time (15–120 min) was studied. A defined mass of the three polymers (0.01 g) was mixed and primary pH was adjusted with 0.1 N NaOH or HCl. The solution was shaken at room temperature for 120 min and then filtered to eliminate any inorganic or organic precipitates formed under basic or acidic conditions. The temperatures were changed by a shaker incubator. The concentration of dye for all the solutions was determined based on the absorbance at a higher wavelength (λ_max_ = 480 nm) via UV–vis spectrophotometry. The dye elimination percentage and sorption capacity were calculated using the following equations [26]:

(1) $$\mathrm{Percentage}\;\mathrm{removal}\;\left(\%\right)=\frac{\left(C_{i}\;-\;C_{f}\right)}{C_{i}}\;\times \;100$$

(2) $$\mathrm{Adsorption}\;\mathrm{capacity}\;\left(q_{e}\right)=\frac{\left(C_{i}\;-\;C_{f}\right)\;\times \;V}{M}\;$$

where C_i_ and C_f_ (mg L^−1^) are the primary concentrations at the initial time and the final concentration MO at a given period, respectively, while V represents the volume of the dye mixture (L), and M is the mass of the composite (g).

### 2.6. Characterization of Nanocomposites

The surfaces of P(AN-*co*-St), SP(AN-*co*-St), and MWCNTs were observed using scanning electron microscopy (JSM 6360 LA, JEOL, Tokyo, Japan) to examine their morphological structures. The composites were investigated at an angle of 11.1° with a particle size analyzer (Beckman Coulter, Miami, FL, USA) to study the distribution of particle sizes by dissolving the composites samples in water solution. Data were examined for 5 min at 20 °C with a refractive index 1.33 and viscosity 0.01 poise [3,14]. FTIR and Raman spectroscopy were used to measure the influence of the prepared polymeric materials via a Shimadzu FTIR-8400 S (Kyoto, Japan) and Senterra Raman spectrometer (Bruker, Billerica, MA, USA), respectively.

### 2.7. Adsorption Equilibrium Isotherm

#### 2.7.1. Langmuir and Freundlich Models

Isothermal models were selected while taking into consideration that the Langmuir model features monolayer sorption on the external surface of the composites (adsorbent), which is based on the supposition that the intermolecular forces decline quickly with distance and can, therefore, predict the presence of monolayer coverage for the sorbate on the external surface of the sorbent. On the other hand, the Freundlich model indicates whether the retention of the dye ions occurs in numerous layers and was used to demonstrate the heterogeneity of the systems and describe the reversible sorption process. The mathematical equations of the Langmuir model and Freundlich model were calculated through the following equations [27,28]:

(3) $$\frac{1}{Q_{e}}\;=\;\frac{1}{{\mathrm{bq}}_{\max}}\;\times \;\frac{1}{C_{e}}\;+\;\frac{1}{q_{\max}}$$

(4) $$q_{e}\;=\;K_{f}C_{e}{{}^{1}}^{/n}$$

(5) $$\log\;q_{e}\;=\;\log\;K_{f}\;+\;\frac{1}{n}\log\;C_{e}$$

where q_e_ is the capacity of adsorption at equilibrium, b is the Langmuir constant, q_max_ is the maximum sorption capacity, K_F_ is the Freundlich constant, and n is the heterogeneity factor.

#### 2.7.2. Temkin Isotherm Model

The Temkin equation considers that the heat of the sorption process linearly reduces with adsorption coverage due to adsorbent–adsorbate interactions, which can be expressed in the following linear equation:

(6) $$q_{e}\;=\;B\ln\;A\;+\;B\ln\;C_{e}$$

where B = (R_T_/b) (J/mol) is the Tempkin constant and related to the heat of adsorption and A represents the equilibrium binding constant (L min^−1^) related to higher binding energy.

#### 2.7.3. The Halsey Isotherm Model

The Halsey model is appropriate for the multilayer sorption process, and fitting of the Halsey equation can contain heteroporous solids [29]. The Halsey model can be applied in the following equation:

(7) $$\ln\;q_{e}\;=\;\frac{1}{n}\ln\;K\;+\;\frac{1}{n}\ln\;C_{e}$$

where n and K are Halsey constants.

## 3. Results and Discussion

### 3.1. Characterization of Adsorbents

#### 3.1.1. FTIR Analysis

**Figure 2:**
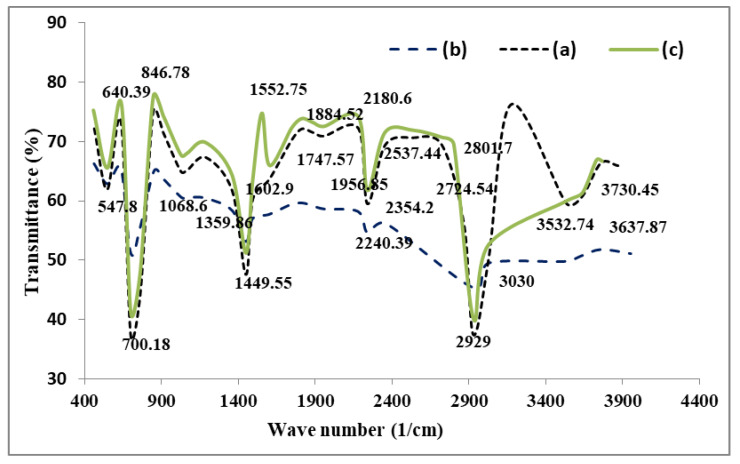
FTIR band assignment of the synthesized P(AN-*co*-St) (**a**), SP(AN-*co*-St) (**b**) and P (AN-*co*-St)/MWCNTs (**c**).

Infrared spectroscopy is a useful instrument for qualitatively delineating the structures of adsorbents. Figure 2a illustrates the spectral property peaks of the functionalized P(AN-*co*-St) nanocomposite. The spectrum that emerged at 1449 cm^−1^ is characteristic of C–C stretching, and the two peaks at 846 and 700.1 cm^−1^ are specific to C–H stretching of the aromatic ring. Appearance of a signal at 1185.3 cm^−1^ is due to aliphatic C–O stretching. Moreover, the peaks in the region at 1333–1552 cm^−1^ correspond to the NH_2_. The band at 1068 cm^−1^ was attributed to the C–N stretching of RNH_2,_ and the sharp band at 700 cm^−1^ is due to the styrene ring [3]. Existence of the peak at 2240 cm^−1^ corresponds to aliphatic C≡N stretching of pure acrylonitrile [4]. Attendance of a band at 2929 cm^−1^ is due to C–H stretching [6]. The bands around 1601–1602 cm^−1^ are due to N–H bending. The signal that emerged at 1449 cm^−1^ is characteristic of C–C stretching. The band at 1747.5 cm^−1^ is similar to that of the carbonyl group (C=O). 

The spectrum of functionalized SP(AN-*co*-ST) (Figure 2) indicates that the peak at 1069 cm^−1^ resembles that of the C–N group of RNH_2_. The bands that emerged at 1746.6 are representative of C=O, close to amine group stretching. Additionally, there is a band at 2355 cm^−1^, indicating thiol S–H stretching. Absorption bands at 3100–3600 cm^−1^ in the spectra of all of the composites were ascribed to carboxylate group stretching caused by the combination of SO_3_H groups. Bands at 2668 to 2966 cm^−1^ are attributed to C–H of alkyls and alkanes.

A sulfonic group is visible in the region of 1040–1197 cm^−1^, which indicates the probability of replacing the (O=S=O) group through the sulfonation method. The sharp band at 761 cm^−1^ is specific to the stretching of the sulfur–carbon [7] and C–H groups of the benzene ring [10].

Figure 2c illustrates the sorption spectra of the MWCNT nanocomposite, where the bands at 3031 and 3521 cm^−1^ are recognized as N–H and O–H stretching, respectively. The peak around 2929 cm^−1^ occurred due to C–H (sp^3^ aliphatic) stretching. Furthermore, the two bands at 1493 cm^−1^ correspond to the carbonyl group (C=O) of the carboxylic group [13].

#### 3.1.2. Scanning Electron Microscopy (SEM) Analysis

**Figure 3:**
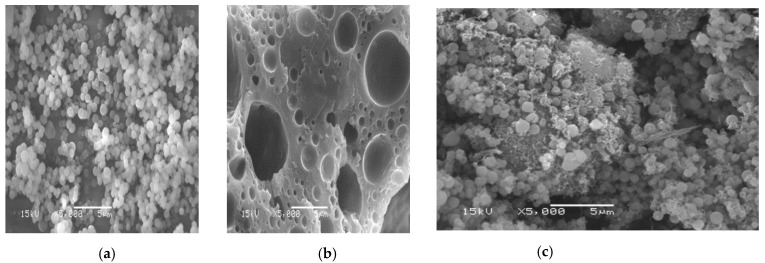
SEM photographs of (**a**) P(AN-*co*-St), (**b**) SP(AN-*co*-St), and (**c**) P(AN-*co*-St)/MWCNTs.

The SEM photographs of the three nanocomposite samples after adsorption are presented in Figure 3. Morphological analysis based on the P(AN-*co*-St) micrograph showed an irregular, homogenous distribution of the spherical nanocomposite and an irregular shape of the polymeric particles forming clusters (Figure 3a), while SP (AN-*co*-St) appeared distorted and displayed a sponge and cave-like structure containing several pores (Figure 3b). The SEM image (Figure 3c) demonstrated that the P(AN-*co*-St)/MWCNTs samples featured certain dimensions, spherical shapes and agglomerated features. These spherical and agglomerated features were determined based on the identified aqueous content and featured intraparticle bonds of the initial poly (acrylonitrile-*co*-styrene) [13].

#### 3.1.3. Particle Size Distribution Analysis (PSD)

Particle size distribution analysis was performed on the three different copolymers with an angle of 11.1°, as shown in Table 2. The data confirm that the unimodal size means were 56.6, 241.4, and 294 nm, whereas the diffusion coefficient was 1.77 × 10^−12^–7.57 × 10^−12^ and the unimodal poly dispersity values were 0.745, −43.616 and −63.915, while count per second ranged between 1.60 × 10^0.06^ and 4.52 × 10^0.05^ for P(AN-*co*-St), SP(AN-*co*-St), and P(AN-*co*-St)/MWCNTs, respectively.

#### 3.1.4. Raman Spectral Analysis

**Figure 4:**
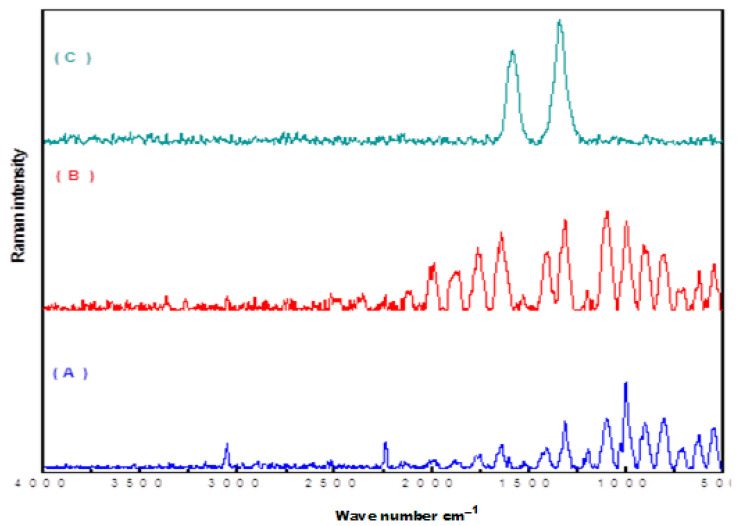
Raman spectrum of (**A**) P(AN-*co*-St), (**B**) SP(AN-*co*-St), and (**C**) P(AN-*co*-St)/MWCNTs.

Raman spectroscopy is usually used as vibrational information is characteristic of the symmetry of particles and chemical bonds, through which the molecule can be identified [2]. The Raman spectra of P(AN-*co*-St), SP(AN-*co*-St), and P(AN-*co*-St)/MWCNTs after the adsorption of dyes are shown in Figure 4 and Table 3. The peaks at 400–450 cm^−1^ are assigned to υ(X metal–O). The absorption peaks at 1000, 1090–1085, and 1360–1380 cm^−1^ are attributed to the υ(C=S) and υ(C–C) aromatic ring chain vibrations and δ(CH3), respectively; the peaks at 1620–1680 cm^−1^ indicate the –C=N– group, while the υ (O–H) and υ(C=O) groups were found at 3100 and 1806 cm^−1^, respectively. The absorption peak at 2244 cm^−1^ is assigned to C–N group stretching.

### 3.2. Optimization of Various Parameters

#### 3.2.1. Initial MO Concentration

**Figure 5:**
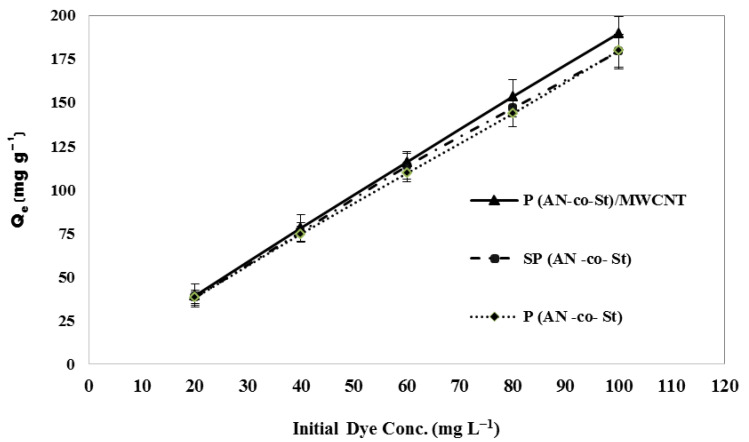
Effect of the initial dye concentration.

The elimination of MO via sorption for the three adsorbent compositions at 20–100 mg L^−1^ of dye at a pH of 5 for 120 min is shown in Figure 5. Adsorption data for a wide range of dye concentrations are the most convenient and relate to adsorption density, q_e_ (dye uptake per unit weight of adsorbent) to equilibrium dye concentration in the bulk of the liquid phase. Figure 5 shows the relationship between the dye uptake all unit weight of adsorbent (q_e_) and the equilibrium dye concentration in the liquid phase (Ce). The amount of dye adsorbed per unit mass of adsorbent increased from 38.10 to 179.88 mg g^−1^ for P(AN-*co*-St), while for SP(AN-*co*-St) it rose from 38.19 to 179.25 mg g^−1^, and increased from 39.23 to 189.77 mg g^−1^ for P(AN-*co*-St)/MWCNTs by increasing the initial dye concentration from 20 to 100 mg L^−1^. This is in agreement with results reported by other studies [30,31,32,33]. The increase in the adsorption capacity is possibly due to greater interaction among the dye and adsorbent in addition to an increase in the driving force of concentration gradient with the increase in the initial concentration. Moreover, the higher amount of dye adsorption at higher concentrations is probably due to increased diffusion and decreased resistance to dye uptake [34].

Bazrafshan et al. [35] showed that the first step of adsorption involves the migration of dye molecules to the outer surface of the P(AN-*co*-St) composite via a liquid film before moving to an internal porous array. Therefore, by increasing the initial dye concentration, an increase in the uptake capacity was accompanied by a decrease in the percentage of removal.

#### 3.2.2. Effect of the Adsorbent Amount on the Percentage of MO Removal

**Figure 6:**
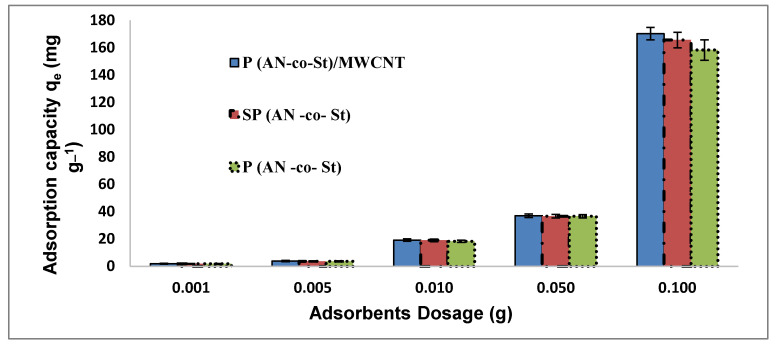
Effect of the adsorbent dosage on the removal process.

Adsorbent dosage is an important factor for the sorption method as it measures the capacity of adsorbents for a given initial dye concentration [36]. A portion of the experiment was performed by changing the polymeric composite dosage upon MO removal to determine how the sorption process could be affected. The removal of MO with a change in composite dosage (0.001–0.1 g) under a 20 mg L^−1^ adsorbate concentration at 30 °C and pH 5 is shown in Figure 6. The results indicate that the adsorption of MO increased from 0.001 to 0.1 g for the three polycomposites, in which MO elimination efficiency is gradually promoted by increased adsorbent. The removal efficiency of P(AN-*co*-St)/MWCNTs was 98.19% for the MO dye solution, agreeing with an uptake capacity of 170.2 mg/g at 0.1 g, while the maximum removal was noticed at 0.1 g with percentages of 94.32% and 96.49%, consistent with adsorption capacities of 158.5 and 165.5 mg g^−1^ by P(AN-*co*-St) and SP(AN-*co*-St), respectively. Increasing the amount of adsorbents led to an increase in the surface area with further functional groups, and more adsorption sites for the adsorbent became obtainable, thereby eliminating more of the dye [1]. An additional increase in the adsorbent amount did not yield any important augmentations in the percentage removal of dyes as the concentration of dyes reached an equilibrium state between the solution phase and solid phase. Furthermore, the interpenetration of the congested active sites occurred at a high adsorbent dose [37]. Additionally, this could be described by the fact that a large number of adsorbents have large surface areas with additional active sites, which improve the penetration method of the dye molecule to the active sites of the adsorbent composite, create it more easily and increasing the ionic interactions [38].

#### 3.2.3. Effect of pH

**Figure 7:**
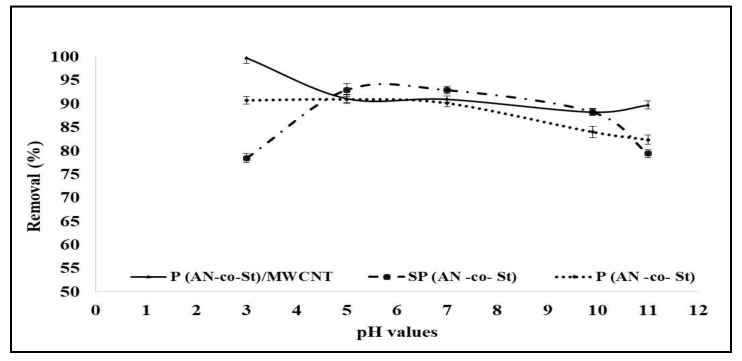
Effect of pH value on the sorption of MO.

The changes in pH values are a vital factor for solute adsorption. These changes control the ionization degree of the adsorbate and thus alter its surface characteristics. Thus, the adsorption capacity of dye is based on the pH of the solution. The effect of serial pH (3–11) on the removal of MO dye for the prepared composites was analyzed under constant conditions (dose of 0.01 g, contact time of 120 min, and 20 mg/L at 30 °C); the results are shown in Figure 7. It can be seen that MO dye removal efficiency increased significantly with a decrease in pH value. Higher adsorption was achieved at pH 5 for both P(AN-*co*-St) and SP(AN-*co*-St), with 90.92% and 92.93% removals, respectively. The maximum removal of MO on P(AN-*co*-St)/MWCNTs, 99.74%, occurred at pH 3. As can be seen from Equations (8) and (9), at a lower pH value (acidic pH), a noticeably high electrostatic affinity was present (electrostatic repulsion) between the positively charged external sites of the poly composites (adsorbents) and anionic dye [37,39]. When the pH value of the adsorption system increased, the number of negatively charged sites increased, while the number of positively charged sites decreased. Moreover, the minimum adsorption efficiency of MO was observed due to the existence of excess soluble hydroxyl ions competing with the dye anions for sorption sites [40].

–C≡N + H–OH ⇌ –C≡N–H(8)

–C≡NH^−^ + Dye^+^ ⇌ –C≡NH^+^ /Dye^−^ (9)

#### 3.2.4. Effect of Contact Time

**Figure 8:**
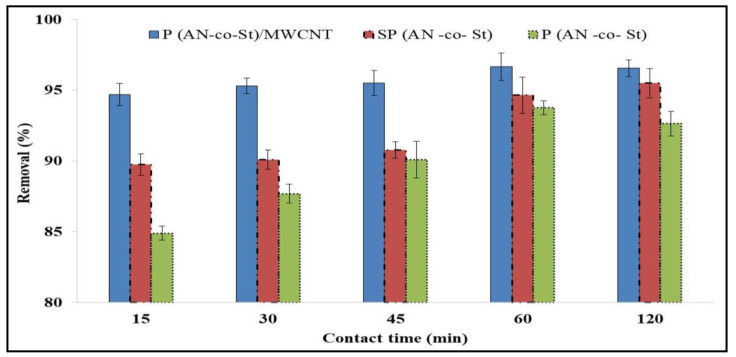
Effect of contact time on the adsorption of MO dye.

The influence of contact time on the removal of MO dye is displayed in Figure 8. The results show that adsorption is fast in the initial stage and adsorption equilibrium is then slowly reached for P(AN-*co*-St). During the first stage, numerous free surface sites exist for the adsorption process, subsequently resulting in the residual surface sites being difficult to occupy due to the phases among repulsive forces [41], in addition to the fact that most of the binding sites were free, which allowed quick binding of ions on the adsorbents.

It can be observed from Figure 8 that the high stability of the adsorption equilibrium of MO was increased from 84.89% to 93.7% and augmented from 94.6% to 96.6% with an increase in contact time from 15 to 60 min for both SP(AN-*co*-St) and P(AN-*co*-St)/MWCNTs, respectively, while contact time was increased from 15 to 120 min, when the adsorption removal of the MO increased from 89.7% to 95.4% on SP(AN-*co*-St).

The most MO was removed within 60 min due to the slow pore diffusion of the solute ion into the bulk of the adsorbent and most of the active sites on the surfaces of the nanocomposites were completely unavailable [42]. Obviously, the quantity of MO dye eliminated by P(AN-co-St)/MWCNTs was not affected by contact time after the early stage. This result is considered as an advantage since it shortened the needed time to generate the functional groups on the adsorbent surface [43]. Moreover, this indicates that the sorption mechanism of P(AN-*co*-St) was completely different from that of SP(AN-*co*-St) and P(AN-*co*-St)/MWCNTs. The spaces available for adsorption in carbon nanotubes are essentially due to the surface site of exterior wall of cylindrical form and not being based on inner wall spacing and the internal cavities [44]. The slow rate of MO sorption after the first hour possibly arose due to the slow pore diffusion of ions of the solute into the majority of the sorbent [9,45]. The sorption rapidly occurred and was normally controlled by the diffusion process from the bulk to the surface. Pick Sheen [46] found that the sorption process was fast in the first 5 min of contact, with removal of further than 90% and equilibrium time was realized in one hour of contact time due to the binding sites of the adsorbent becoming exhausted, the removal percentage slowed down owing to competition for decreasing obtainability of actives sites via ions. Consistent with the experiment results, agitation time was fixed at two hours for the rest of the batch experimentation to confirm that equilibrium time was achieved. In technique application, this fast (or rapid) adsorption phenomenon is valuable as the shorter contact time efficiently allows for a smaller size of the contact apparatus, which in turn directly affects both the operation cost and capacity of the technique.

#### 3.2.5. Effect of Temperature

**Figure 9:**
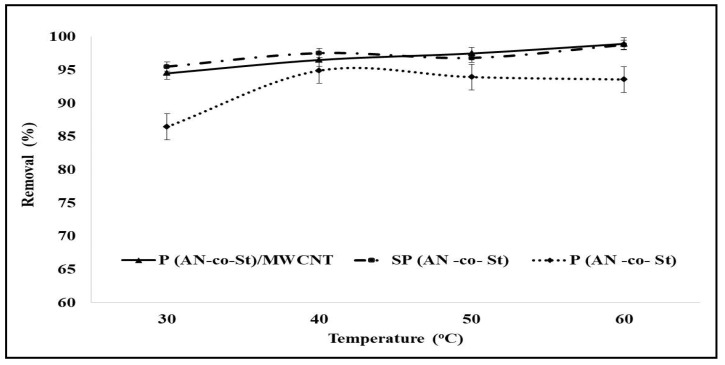
Effect of temperature on the adsorption of MO.

The adsorption process is dependent on temperature, which is associated with numerous thermodynamic parameters. Figure 9 demonstrates the influence of different temperature parameters on the MO removal percentages of three nanocomposites. Figure 9 illustrates a small increase in destination with a temperature increase from 30 to 40 °C for P(AN-*co*-St); after 40 °C, the percentage removal slightly decreased, which indicates that physical adsorption is usually exothermic. This behavior may be due to the MO dye removal method not requiring extra costs or additional heating. Moreover, this behavior may be caused by the acceleration influences of temperature on the particle sorption of MO dyes on the surface of the adsorbent particles [6]. Volesky [47] showed that the binding of initial concentration through the adsorbent was augmented from 50% to 70% after raising the temperature; when the temperature was raised to 40 °C, the binding of the initial concentration was augmented, whereas temperatures of 60 °C or over caused an alteration in the loss of the capacity of adsorption and the texture of the adsorbent.

Conversely, Figure 9 demonstrates that the percentage of MO dye removal increased with a temperature increase for SP(AN-*co*-St) and P(AN-*co*-St)/MWCNTs, which suggests an endothermic nature for the sorption. As the temperature increases, the possible number of active sites increases and the boundary layer of adsorbents decreases, thereby increasing the adsorption [48]. Furthermore, at a high temperature, the rate of activation of functional groups on the surface of the composite polymer increased and thus the efficiency of the polymer on adsorption increased, as the polymer can withstand temperatures of up to 500 °C.

The rapid adsorption in the early stage decreases the concentration gradient between the liquid phase (MO dye) and solid phase (copolymer). The concentration of MO restrictions on one side and the high concentration of exchange sites with molecule surfaces on the other side contribute substantially to this behavior. Moreover, the deficiency of the pore diffusion technique reduces the effect of temperature. Eldin et al. [49] reported that poly acrylonitrile particles have no greater effect after increasing the temperature to 60 °C. This might be a result of wholly dissociating the available initiator, ensuring that no extra free radicals are able to be produced due to an increase in temperature [50]. Greene and Darnall [51] showed that the ratio of distribution between bound ions/ions in solution for adsorption of some metal ions to adsorbent augmented by only ~20% when the temperature range was high, from 4–55 °C, which shows the influence of temperature is minor as compared to other influencing parameters. 

### 3.3. Adsorption Isotherm Studies

Sorption isotherms offer qualitative data on the nature of the solute–external surface interactions and adsorbent capacity [52]. In this study, modeling of the investigational isotherms, starting with the adsorption of dyes on adsorbent materials, was performed using the Langmuir and Freundlich and Halsey and Temkin isotherm models to demonstrate the equilibrium features of sorption and to understand the behavior of the adsorption isotherm model with the investigational data. The theoretic Langmuir adsorption isotherm is the most widely used for the adsorption of a pollutant from an aquatic solution. It is valid for removal of a solute from a liquid mixture as monolayer adsorption on a finite number of identical sites (specific homogenous sites) within the adsorbent external, which are energetically equivalent [52].

**Figure 10:**
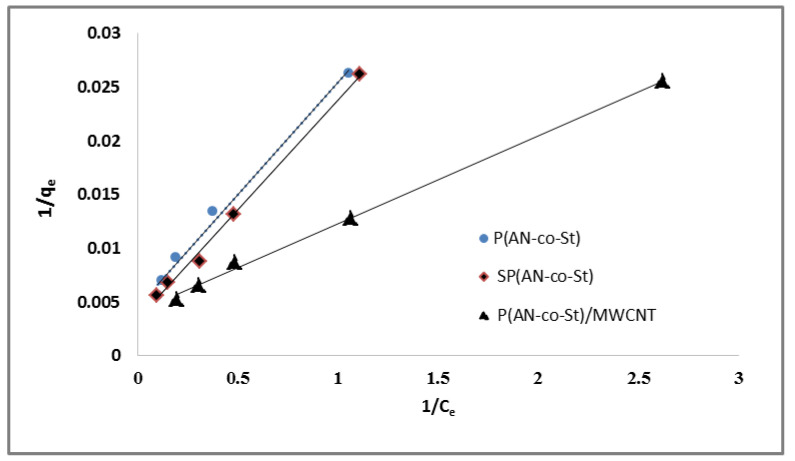
Langmuir plots for adsorption of MO onto different composites.

The linear demonstrations of the Langmuir models (1/q_e_ vs. 1/c_e_) for the removal of MO dye on three nanocomposites, which provided straight lines for 1/q_max_ (slope) and 1 /q_max_ b (intercept), are illustrated in Figure 10, and the isotherm factors calculated for all composites are presented in Table 4. The values of parameters Q _max_ (mg g^−1^) and b were calculated from the slope and intercept of the plot, respectively. The data for MO dye removal onto the P(AN-*co*-St), SP(AN-*co*-St), and P(AN-*co*-St)/MWCNTs nanocomposites were entered into the Langmuir equation with the linear correlation coefficient R^2^ = 0.990, 0.996, and 0.998, respectively. The constants of Langmuir “q_max_” and “b” were determined by the maximum sorption capacity and energy of the sorption process. From the results, it was showed that the maximum MO uptake (q_max_) values were 121.95, 48.78 and 47.84 mg g^−1^ for P(AN-*co*-St), SP(AN-*co*-St), and P(AN-*co*-St)/MWCNTs, respectively. The P(AN-*co*-St)/MWCNTs composite has a higher adsorption capacity than P(AN-*co*-St), and SP(AN-*co*-St). The Langmuir constant (b), which is related to the heat of adsorption of MO, was found to be 4.52, 6.21 and 2 by P(AN-*co*-St), SP(AN-*co*-St), and P(AN-*co*-St)/MWCNTs, respectively.

The strong applicability of the Langmuir isotherm equation to this adsorption process shows that this model is suitable for homogeneous adsorption, for which the adsorption processes of all dye particles onto external surfaces are equal to the energy of adsorption activation. The value of q_e_ increases with the augment in initial MO concentration; this is due to the higher availability of MO dye to adsorb at a higher initial concentration of MO dye.

The Langmuir equation was used to calculate the affinity of the adsorbent surfaces to dye based on the R_L_ values (dimensionless separation factor) determined via Equation (10):

R_L_ = 1/(1 + b × C_i_) (10)

**Figure 11:**
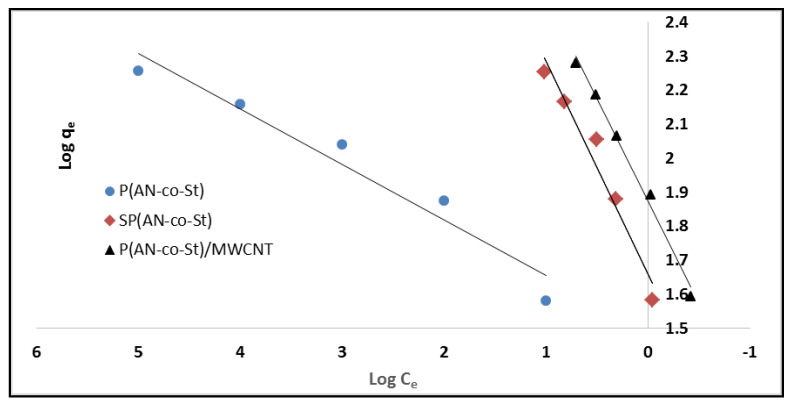
Freundlich plots for adsorption of MO onto different composites.

The average R_L_ values were 0.00499, 0.0036, and 0.011 for P(AN-*co*-St), SP(AN-*co*-St), and P(AN-*co*-St)/MWCNTs, respectively, which means that the adsorption process was favorable as the values of R_L_ are between 0 and 1, as reported by Langmuir [27]. Freundlich isotherm considered the sorption of the dye onto the heterogeneous external of an adsorbent. The Freundlich constants of K_f_ and n parameters represent the sorption capacity and sorption intensity, respectively, which can be expressed by the figure of log (q_e_) vs. log (C_e_), as presented in Figure 11 and Table 4. The “K_f_” values were calculated to be 30.9, 45.6 and 63.09, while “n” values were determined to be 6.1, 1.6 and 1.66 for P(AN-*co*-St), SP(AN-*co*-St), and P(AN-*co*-St)/MWCNTs, respectively. If the values of parameter “n” are between 1 and 10 [53], the data for this model indicate a favorable adsorption process on the surface. The coefficient values achieved using the Freundlich model was obtained for three nanocomposites (Table 4). Freundlich isotherm fitted well with the correlation coefficients of 0.947, 0.961 and 0.991 for P(AN-co-St), SP(AN-*co*-St), and P(AN-*co*-St)/MWCNTs, respectively.

**Figure 12:**
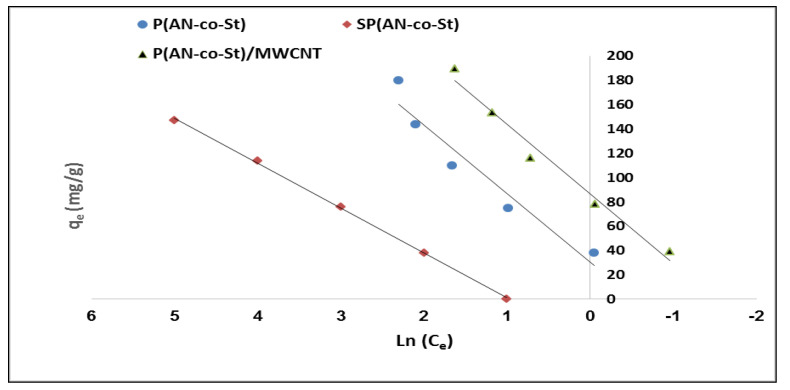
Temkin plots for adsorption of MO onto different composites.

**Figure 13:**
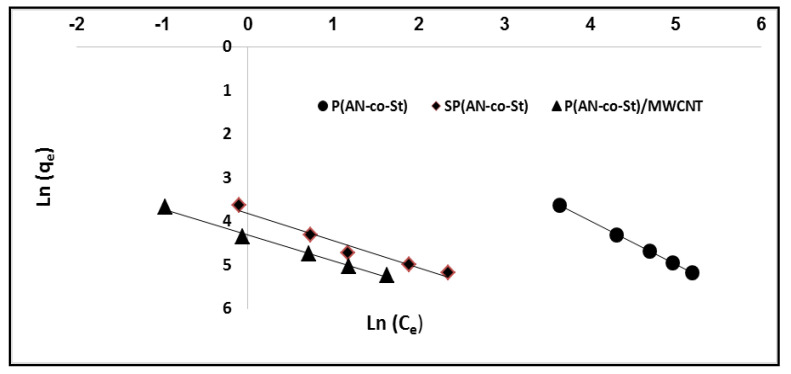
Halsey plots for removal of MO onto different composites.

The Temkin isotherm model assumes that the heat of the sorption process of all of the molecules would decrease linearly. Consequently, the linear plots of q_e_ vs. ln C_e_ allowed us to measure the Temkin isotherm parameters K_T_ and b_T_ from the slope and intercept, respectively, as presented in Figure 12 and Table 4. The data indicate that the Temkin isotherm model is appropriate for the adsorption of MO dye onto three adsorbents, as shown by the high values of the linear regression correlation coefficient, which were determined as 0.931, 0.999 and 0.977 for P(AN-*co*-St), SP(AN-*co*-St), and P(AN-*co*-St)/MWCNTs, respectively. The Halsey isotherm equation is appropriate for the multilayer adsorption process and heteroporous solids. The plots of ln q_e_ against the ln Ce Halsey adsorption isotherms are shown in Figure 13. The parameters obtained for the Halsey isotherm were fitted with P(AN-*co*-St), SP(AN-*co*-St), and P(AN-*co*-St)/MWCNTs, with high regression correlation coefficients ranging between 0.961 and 0.996. This indicates that the Halsey isotherm are applicable to the adsorption of MO onto adsorbents. The Halsey isotherm parameters of K and n are presented in Table 4. The best fit isotherm models nearly followed the order: Halsey, Langmuir, Freundlich and Temkin isotherm models for P(AN-*co*-St). Moreover, the Temkin, Langmuir, Freundlich and Halsey models were appropriately fitted for SP(AN-*co*-St), while the applicability of the isotherm models for the P(AN-*co*-St)/MWCNTs was Langmuir, Temkin, Freundlich and Halsey isotherms.

Among the nanocomposites examined, P(AN-*co*-St)/MWCNTs have shown the highest adsorption capacity for MO (qmax =121.95 mg g^−1^). These were followed by SP(AN-*co*-St) with a qmax = (48.78 mg g^−1^) and P(AN-*co*-St) with a qmax = (47.84 mg g^−1^), respectively. 

#### Comparative Analysis of the Sorption Capacity of Different Composites 

The results of the present study were compared with those of other works on the adsorption capacities of various dyes into polyacrylonitrile, modified acrylonitrile, and MWCNTs (Table 5).

### 3.4. Kinetic Study of MO Adsorption Process (Adsorbent Rate Constant) 

Sorption kinetic equations were used to describe the mechanisms and characteristics of adsorption [45]. Consistent with the literature, Ho’s pseudo-first- and second-order equations (from Lagergren Ho) were used to include the adsorption mechanism [58]. The Lagergren pseudo-first-order (PFO) and Ho’s pseudo-second-order (PSO) models are represented through the following linear equations:

Log (q_e_ − q_t_) = log q_e_ − (k_1_/2.303)t (11)

T/q_t_ = 1/K_2_ q_e_2 + (1/q_e_)t (3) (12)

where q_e_ and q_t_ (mg g^−1^) are the quantities of adsorbed dye at equilibrium and instant time, respectively; k_1_ (l min^−1^) is the rate constant of adsorption (1 min^−1^); and K_2_ (g mg^−1^ min^−1^) is the second-order rate constant. 

**Figure 14:**
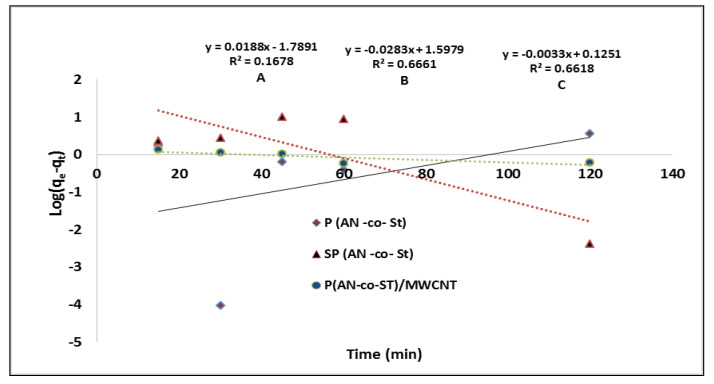
First-order kinetics for the removal of MO onto various composites.

**Figure 15:**
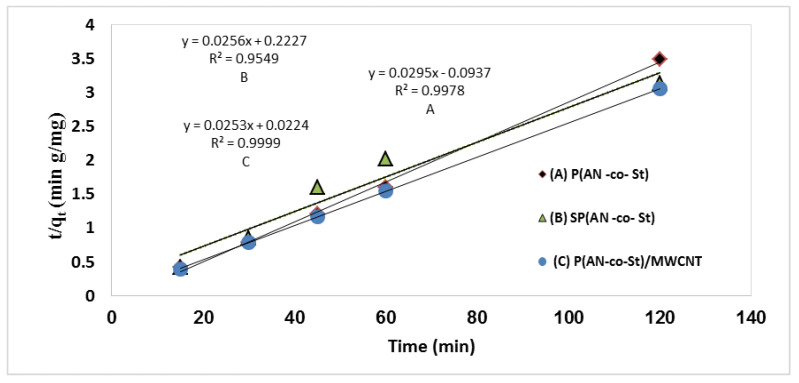
Second-order kinetics for the removal of MO onto various composites.

The plot of the value log (q_e_ – q_t_) vs. (t) should provide a linear correlation form, where the predicted *q_e_* and *k_1_* values can be calculated from the intercept and slope of the linear relationship of the plots, respectively (Figure 14). The intercepts and slopes of the plots were used to determine the values of the parameters “k_2_” and “q_e_” based on the relation between (t q_t_^−1^) vs. (T) for the second-order kinetics. The values of linear correlation coefficient of pseudo-first-order equations are 0.166, 0.666 and 0.661 for P(AN-*co*-St), SP(AN-*co*-St) and P(AN-*co*-St)/MWCNTs, respectively. The data obtained in this study were not gained by employing the first-order model due to the low linear regression correlation coefficient. This recommends that this sorption process does not undergo a first-order reaction. Nevertheless, the experimental results showed good correlation equal to unity, indicating that the sorption kinetics of MO favor the second-order model, as shown in Table 6 and Figure 15. The factors of the first- and second-order models listed in Table 6 demonstrate a good determination coefficient value (0.997) for P(AN-*co*-St), while this value was 0.997 and 0.999 for SP(AN-*co*-St) and P(AN-*co*-St)/MWCNTs, respectively. Moreover, it was noticeable that the values of k_2_ were greater than the consistent values of k_1_ in all results. This was due to the pseudo-second-order kinetic model assumed that the adsorption rate is proportional to the square of a number of unoccupied sites [59]. The observed results showed that the best fit kinetic model was pseudo-second-order equation for adsorption of MO dye by three adsorbents. This model has been used in numerous studies in this field, as reported by Elzain et al. [1] and Eldin et al. [60].

### 3.5. Reusability Study of MO Dye

**Figure 16:**
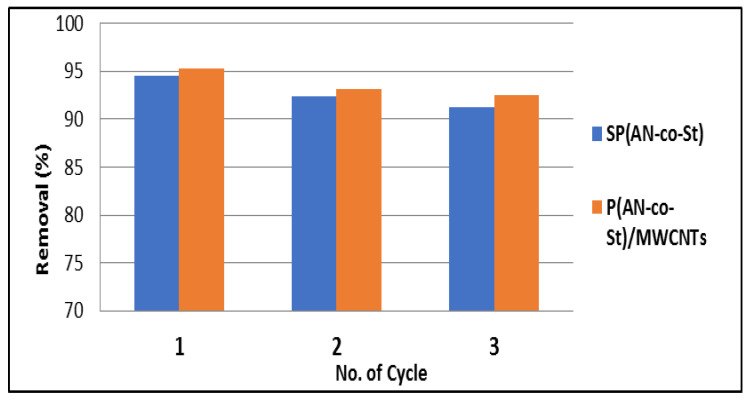
Reusability study of MO dye by SP(AN-*co*-St) and P(AN-*co*-St)/MWCNTs.

Regeneration studies on the probability of desorbing MO molecules from SP(AN-*co*-St) and P(AN-*co*-St)/MWCNTs are useful for industrial and economic applications. The results showed that the reuse of SP(AN-*co*-St) and P(AN-*co*-St)/MWCNTs for MO dye remains nearly unchanged for three consecutive cycles. The initial removal efficiency values were 94.55% and 95.34%, which reduced slightly to 91.2% and 92.46% for SP(AN-*co*-St) and P(AN-*co*-St)/MWCNTs, respectively, after the third round, as shown in Figure 16. Hence, it could be concluded that the adsorption capacity of MCNTs stays unaffected with extended regeneration series.

## 4. Conclusions

The current research focused on estimating the enhancing influence of incorporating P(AN-*co*-St) materials into MWCNTs and the sulfonic group’s adsorptive capacity toward cationic methyl orange dye. The poly (acrylonitrile–*co*–styrene) nanocomposite was prepared via a simple precipitation polymerization process. MWCNTs were produced using the CVD process with a tube furnace with a 60 cm length quartz tube and a 45 mm diameter. The adsorption of the MO dye from an aqueous solution was tested for three different nanocomposites. The influence of some factors was assessed by a batch experiment, comprising initial MO concentration (20–100 mg L^−1^), pH (3–11), contact time (15–120 min), adsorbent amount (0.001–0.1 g), and temperature (30–60 °C) during the adsorption process. In these tests, 60 min of contact time at pH values of 3 and 5 was found to be adequate for eliminating over 90% of 20 mg L^−1^ MO at 30 °C composites in the adsorption of the high methyl orange concentration, with a greater rate of sorption. The kinetics of MO sorption were not appropriate for first-order kinetics but were very appropriate for second-order kinetic models, as the linear correlation coefficient almost reached unity. The maximum sorption capacity for the P(AN-*co*-St)/MWCNTs was 121.95 mg g^−1^, which is higher when compared with data provided in previous studies. The Langmuir, Freundlich, Temkin, and Halsey isotherms were appropriate for modeling the equilibrium results of the three adsorbents. The structures of P(AN-*co*-St) composites were confirmed by Raman and FTIR analyses. This could have resulted from the FTIR spectra of the functional groups existing in the adsorbents being responsible for the MO uptake such as NH_2_, C–N, C≡N, C=O, thiol S–H, O–H and O=S=O. The morphological structures of the nanocomposite molecules were indicated via particle size distribution and scanning electron microscope photographs. SEM images display the irregular, homogenous distribution of the spherical shapes of the nanocomposites. Ultimately, these composites can be possibly used as efficient materials for the adsorption of MO dye from aqueous solutions, and P(AN-co-St)/MWCNT was identified as the most promising adsorbent due to its high uptake.

## Figures and Tables

**Table 1:** Properties of methyl orange dye.

Chemical Formula	C_15_H_15_N_3_O_2_
Molecular mass (g mol^−1^)	269.30 g mol^−1^
Maximum wavelength	480 nm
Uses	Textile
Melting point	179–182 °C
Density	0.791 g cm^3^
pH	pH 4.2: pink, pH 5.5: orange pH 6.2: yellow
Molecular Structure	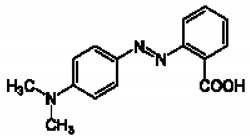

**Table 2:** Particle size distribution analysis of nanocomposites.

Polymer	Angle	Mean (nm)	P.L	Diff. Coef (m² s^−1^)	Counts/s	Baseline Error
P(AN-*co*-St)	11.1°	56.6	0.745	7.57 × 10^−^^12^	1.60 × 10^0.06^	2.84%
SP(AN-*co*-St)		241.4	−43.616	1.77× 10^−^^12^	3.59 × 10^0.06^	0.29%
P(AN-*co*-St)/MWCNTs		294.0	−63.915	4.31 × 10^−^^13^	4.52 × 10^0.05^	1.87%

**Table 3:** Raman spectra showing functional group vibration for different nanocomposites after the adsorption of dyes.

Materials	Wavenumber (cm^−1^)	Assignment
P (AN-*co*-St)	410	υ(Xmetal–O)
	650	υ(C–S) aliphatic
	810, 965	υ(C–O–C)
	1090 (w)	υ(C–S) aromatic
	1070, 1150	υ(C=S)
	1450 (w)	υ(C–C) aromatic ring
	1360 (w)	δ(CH_3_)
	1680 (w)	υ(C=N)
SP(AN-*co*-St)	410	υ(Xmetal–O)
	1000, 1100, 1220 (w)	υ(C=S)
	1650	υ(C=N)
	2240	υ(C≡C)
	2850	υ(C−H)
P(AN-*co*-St)/MWCNT	410	υ(Xmetal–O)
	640, 720	υ(C–S) aliphatic
	830, 910	υ(C–O–C)
	1085	υ(C–S) aromatic
	1000	υ(C=S)
	1000	υ(C–C) aromatic ring
	1380	δ(CH_3_)
	1620	υ(C=N)
	1750	υ(C=C)
	1806	υ(C=O)
	2000	υ(C≡C)
	3100	υ(O–H)

**Table 4:** Isotherm parameters for the removal of MO dye on different nanocomposites.

Isotherm Model	Parameters	P(AN-*co*-St)	SP(AN-*co*-St)	P(AN-*co*-St)/MWCNTs
Langmuir	R^2^	0.990	0.996	0.998
	q_max_ (mg g^−1^)	47.84	48.78	121.95
	b (L mg^−1^)	4.54	6.21	2
	R_L_	0.00499	0.0036	0.011
Freundlich	R^2^	0.947	0.961	0.991
	n	6.1	1.6	1.66
	K_f_ (mg g^−1^)	30.9	45.6	63.09
Temkin	R^2^	0.931	0.999	0.977
	A (L g^−1^)	17.63	0.97	3.85
	B(mg L^−1^)	56.23	36.89	57.1
	b_T_	0.0005	0.00076	0.00049
Halsey isotherm	R^2^	0.996	0.961	0.991
	1/n	1.565	0.625	123.84
	K	196.7	72.86	0.601

**Table 5:** Summary of the elimination of dye from aqueous solutions by P(AN-*co*-St) and different carbon nanotubes.

Adsorbent Used for Adsorption	Dye	Sorption Capacity (mg g^−1^)	References
Poly (AN-*co*-St) NFs	Methylene blue	15.84	[1]
MWCNTs	Malachite green	142.85	[17]
MWCNTs	Methyl orange	18.95	[54]
Carbon nanotubes	Methylene blue	35.4	[21]
Carbon nanotubes	Methylene blue	103.62	[55]
Nano poly acrylonitrile particles	Methylene Blue	8.7600	[43]
MWCNTs	Methyl orange	5.181	[56]
Poly (AN-*co*-St)/graphite NFs	Methylene blue	18.73	[1]
GO hydrogel	Rhodamine B (RhB),	7.85	[57]
Poly (AN-*co*-St)/CNTs NFs	Methylene blue	23.55	[1]
Poly iminated polyacrylonitrile	Methylene Blue	54	[34]
P(AN-*co*-St)	Methyl orange	47.84	This study
SP(AN-*co*-St)	Methyl orange	48.78	This study
P(AN-*co*-St)/MWCNTs	Methyl orange	121.95	This study

**Table 6:** Parameters for adsorption kinetics.

Model	1st-Order Kinetic Model			2nd-Order Kinetic Model		
Parameters	R^2^	k_1_ (1 min^−1^)	q_e_ (calc.) (mg g^−1^)	R^2^	k_2_ (g mg^−1^ min^−1^)	q_e_ (calc.) (mg g^−1^)
P(AN-*co*-St)	0.167	0.0432 ± 0.01	61.53 ± 5.67	0.997	347.23 ± 23.48	0.00288 ± 0.001
SP(AN-*co*-St)	0.67	0.07 ± 0.01	39.62 ± 7.01	0.997	0.01 ± 0.001	107.67 ± 12.02
P(AN-*co*-St)/MWCNT	0.66	7.599 × 10^−3^ ± 91.00	1.33 ± 0.21	0.999	1768.05 ± 86.00	0.00056 ± 0.00

## Data Availability

All relevant data are within the paper and are available from the corresponding author.
